# Childhood cumulative trauma, social support and stress as predictors of illness outcomes and quality of life in bipolar disorder

**DOI:** 10.1177/00048674231209225

**Published:** 2023-11-09

**Authors:** Amy-Leigh Rowe, Tania Perich, Tanya Meade

**Affiliations:** 1 School of Psychology, Western Sydney University, Penrith, NSW, Australia; 2 Translational Health Research Institute, Western Sydney University, Penrith, NSW, Australia

**Keywords:** Trauma, stress, social support, bipolar disorder

## Abstract

**Objective:**

Trauma, social support and stress have been identified as factors which may be associated with the bipolar disorder illness course. However, these are yet to be examined in prospective studies as predictors of illness outcomes and overall quality of life in bipolar disorder.

**Method:**

One hundred and fourteen participants (*N* = 97; 85.1% female) living with bipolar disorder I (41.2%) or II (58.8%) completed a semi-structured interview and a range of self-report measures assessing trauma history, social support, perceived stress, depression, anxiety, mania, suicidality, number of mood episodes and quality of life, at baseline and 6-month follow-up.

**Results:**

Childhood cumulative trauma, social support and perceived stress accounted for a substantial and significant portion of the variance in quality of life (62%; 23.6%), anxiety severity (34.6%; 24.5%) and depression severity (49.6%; 26.7%), at both baseline and 6-month follow-up. Perceived stress made significant unique contributions to the prediction of all outcomes, and social support made significant unique contributions to depression and quality of life in bipolar disorder.

**Conclusion:**

Stress and social support play an important role in bipolar disorder and in quality of life for people living with this condition. Given that stress and social support are modifiable risk factors, this provides a promising direction for future intervention-based research.

## Introduction

Bipolar disorder (BD) is a chronic psychiatric condition characterised by recurrent mood episodes interspersed with periods of remission. Over the course of the condition, those with BD spend on average half of their time symptomatic (Hirschfeld, 2014). BD is associated with a range of adverse outcomes, including impaired functioning (Elias et al., 2017) and reduced quality of life (Michalak et al., 2005; Morton et al., 2018). In Australia, the economic burden of BD represents one of the highest of all mental disorders at $7.39 billion per annum, comprising 39% of total public expenditure on mental health (KPMG, 2014).

Rates of trauma history are much higher amongst those with BD than the general population (Hernandez et al., 2013). Early trauma exposure in BD is associated with a more severe clinical presentation and a decreased ability to cope with later stressors (Aas et al., 2016), as well as an increased risk of substance use and suicide (Aas et al., 2016; Daruy-Filho et al., 2011; Dualibe and Osório, 2017; Etain et al., 2013; Maniglio, 2013). Exposure to cumulative trauma during childhood may add further complexity to the clinical picture. For example, in non-BD samples, associations have been demonstrated between exposure to multiple trauma types during childhood and ongoing symptoms of depression and dysthymia (Martin et al., 2013; Suliman et al., 2009), dissociation (Martin et al., 2013) and emotion regulation difficulties (Konecky and Lynch, 2019). Despite this, little is known about the role of childhood cumulative trauma for people living with BD.

Social support is often heralded as a protective factor due to its association with better mental health outcomes (Bjørlykhaug et al., 2022; Harandi et al., 2017). However, in comparison to the general population, those with BD experience lower levels of social support (Uygun et al., 2020; Warren and Fowler, 2021), and those with a combination of BD and trauma report even lower perceptions of social support (Zhou et al., 2019). Studies examining social support in BD have focused primarily on the severity of or recurrence of mania and depression (Eidelman et al., 2012; Koenders et al., 2015; Wang et al., 2018) where it has been found that lower levels of perceived social support significantly predicted greater depression scores over time, lower levels of functioning and, among those with BD-I, a greater risk of manic or depressive recurrence. One study has found that social support mediates the relationship between childhood trauma and adult depressive symptoms (Zhou et al., 2019), suggesting a unique role for social support in the course of the illness.

Stress is another important factor that warrants consideration. For example, in their prospective study involving a sample of outpatients with BD-I who were followed up every 3 months for 1 year, Cohen et al. (2004) found that higher levels of stress and lower levels of perceived social support were associated with depressive, but not manic recurrence. Furthermore, an investigation of the trauma and stress relationship in BD found that lifetime trauma exposure is associated with increased severity of interpersonal chronic stressors, and 7.5% of the variance in depressive symptoms can be attributed to overall chronic stressors (Gershon et al., 2013).

General population studies may also provide more insight into the unique roles of early trauma, social support and stress. For example, Mc Elroy and Hevey (2014) investigated the relationship between early trauma, stressors and psychosocial resources such as social support and wellbeing. Their study found that with the exception of physical abuse and death of a parent, all other early adverse experiences were associated with exposure to a greater number of stressors and decreased wellbeing (Mc Elroy and Hevey, 2014). In addition, they found that the relationship between number of early adverse experiences and overall wellbeing was partially mediated by the number of stressors (Mc Elroy and Hevey, 2014).

Some studies have highlighted the links between trauma and stress (Gershon et al., 2013), and social support and stress have been identified as significant predictors of depressive recurrence (Cohen et al., 2004). However, the three variables (cumulative trauma, social support and stress) have never been assessed for their combined ability to predict prospective features of the bipolar illness or quality of life. For lifelong conditions such as BD, including a quality of life measure is of particular importance, as it provides insight into experiences of wellbeing, beyond the presence or absence of illness symptoms (Michalak and Murray, 2010a).

Therefore, this study aimed to determine whether childhood cumulative trauma, perceived social support and perceived stress predict BD illness outcomes and quality of life. It is hypothesised that at baseline, higher childhood cumulative trauma scores, lower perceived social support and greater perceived stress will be significant predictors of greater BD symptom severity and lower quality of life at baseline and 6-month follow-up.

## Method

### Participants

Participants comprised 114 people (*N* = 97; 85.1% female) living with BD-I (41.2%) or BD-II (58.8%) from six countries: Australia (*n* = 38), America (*n* = 34), South Africa (*n* = 25), Canada (*n* = 11), United Kingdom (*n* = 4) and New Zealand (*n* = 2). All participant demographics and clinical characteristics are presented in Table 1. Inclusion criteria specified that participants must (a) be over 18 years of age, (b) have received a primary diagnosis of BD-I or BD-II and (c) currently be under the care of a general practitioner (GP) or psychiatrist. Exclusion criteria were (a) a diagnosis of cyclothymia, (b) current active suicidal ideation and (c) not under the care of a GP or psychiatrist. This study received ethics approval from the Human Research Ethics Committee at Western Sydney University (H13572).

**Table 1.** Demographic and clinical characteristics of the sample at baseline.

	*n*	%
Country of residence		
Australia	38	33
America	34	30
South Africa	25	22
Canada	11	10
United Kingdom	4	4
New Zealand	2	2
Sex		
Female	97	85
Male	17	15
BD subtype		
I	47	41
II	67	59
Marital status		
Partnered	47	41
Not partnered	67	59
Employment		
Yes	65	57
No	49	43
Government benefits		
Yes	45	39
No	69	61
Last episode hospitalised		
Yes	40	35
No	74	65
Past suicide attempt		
Yes	59	52
No	55	48
Rapid cycling (lifetime)		
Yes	36	32
No	78	68
Mixed mood (lifetime)		
Yes	52	46
No	62	54
Currently symptomatic		
Yes	64	56
No	50	44
Education level		
Secondary schooling	23	20
Vocational qualification	34	30
Bachelor’s degree or higher	57	50
Hospitalisations (lifetime)		
0–2	54	47
3–5	37	32
6+	23	20
Other mental health diagnosis (lifetime)		
Yes	88	77
No	26	23

BD: bipolar disorder.

*N* = 114. All % reported to the nearest whole number.

### Procedure

Participants were recruited from 8 July 2020 until 30 October 2020 via Facebook paid advertising, which was set up to be displayed only to people who were living in predominantly English-speaking countries and who had shown interest in a range of mental health organisations (Beyond Blue, Depression and Bipolar Support Alliance, Anxiety and Depression Association of America, National Alliance of Mental Illness, International Bipolar Foundation, National Institute of Mental Health, National Suicide Prevention Lifeline). Additional recruitment took place via advertising on the Western Sydney University website. In order to meet recruitment targets and in line with similar studies in this area (Baldessarini et al., 2012; Huesch et al., 2018; Perich and Ussher, 2022), an international approach to convenience sampling was sought. Participants provided written informed consent prior to involvement in the study. Reimbursement was given to 20% of the participants who were randomly selected to receive a $50 e-gift card.

Participants first completed an online survey via Qualtrics which assessed demographic details, childhood trauma history, bipolar symptoms, quality of life, social support and perceptions of stress. Participants were then interviewed via phone by the first author (A-L.R.) to confirm their BD diagnosis using the Structured Clinical Interview for Diagnostic and Statistical Manual of Mental Disorders, Fifth Edition, (*DSM*-5) (SCID-5-RV). Participants completed all self-report measures via Qualtrics at baseline and 6-month follow-up, except for the trauma history measure (*Childhood Trauma Questionnaire* [CTQ]) which was only completed at baseline. Participants also completed clinical interviews at both timepoints, all of which were conducted by the lead researcher (A.-L.R.). Mania and depression symptoms were assessed using the Young Mania Rating Scale (YMRS) and the Montgomery Asberg Depression Rating Scale (MADRS). Interviews took place via phone call, within 2 weeks of survey completion. At the 6-month follow-up interview, the SCID-5-RV was modified to determine the type and number of mood episodes experienced in the previous 6 months, whereby only the criteria for a major depressive, hypomanic and manic episodes were used.

### Measures

#### Researcher administered

##### Montgomery-Asberg Depression Rating Scale

The MADRS is a 10-item clinician-administered scale which is used to evaluate the severity of depression symptoms within the last 7 days (Montgomery and Åsberg, 1979). Each item is rated from 0 to 6, with 6 representing the most depressed mood. The MADRS has good inter-rater reliability (0.76) (Davidson et al., 1986) and demonstrated excellent internal consistency in the present study (α = 0.913).

##### Young Mania Rating Scale

The YMRS is an 11-item clinician-administered scale used to identify symptoms of mania (Young et al., 1978). A rating of 0 (no symptoms) to 4 (extreme deviation) is made by the clinicians based on the persons’ account of their experience in the last 48 hours. Total scores range from 0 to 44, with higher scores indicating greater mania severity. The YMRS has demonstrated good inter-rater reliability with correlations as high as 0.95 (Young et al., 1978). The scale demonstrated good internal consistency in the present study (α = 0.817).

##### Structured Clinical Interview for *DSM*-5 Research Version

The SCID-5-RV is a researcher-administered interview schedule designed to assess symptoms of a number of psychiatric conditions, including BP. Current and lifetime symptoms of BP (depression, hypomania, mania, psychosis) were assessed, as well as history of mixed episodes, rapid cycling, frequency of episodes and frequency of hospitalisations. Diagnostic reliability has been reported with good internal consistency (Cronbach’s α > 0.80), convergent validity and test–retest reliability (Shankman et al., 2018). The SCID-5-RV was modified for 6-month follow-up interviews whereby only criteria for major depressive, hypomanic and manic episodes were used to determine the presence of such episodes over the previous 12 weeks.

#### Self-report measures

##### Childhood Trauma Questionnaire

The CTQ is a 28-item retrospective self-report measure which examines the first 17 years of life, specifically measuring the frequency and severity of three types of abuse (physical, emotional and sexual) and two types of neglect (physical and emotional) (Bernstein et al., 1998). Items are rated on a 5-point Likert-type scale from 1 (never true) to 5 (very often true). Total scores for each subscale range from 5 to 25, with higher scores indicating a greater exposure to trauma. In the present study, all subscales demonstrated good internal consistency: physical neglect (α = 0.818), emotional neglect (α = 0.933), emotional abuse (α = 0.891), sexual abuse (α = 0.953) and physical abuse (α = 0.902).

##### Multidimensional Scale of Perceived Social Support

The Multidimensional Scale of Perceived Social Support (MSPSS) is a 12-item self-report scale designed to assess feelings of perceived social support (Zimet et al., 1988). Items are rated on a 7-point Likert-type scale from 1 (strongly disagree) to 7 (very strongly agree), with a total possible score ranging from 12 to 84, whereby higher scores are indicative of feeling more supported by the social environment (Zimet et al., 1988). Items span three domains of support: family, friends and significant other. Each of the subscales demonstrated excellent internal consistency in the present study: significant other (α = 0.951), family (α = 0.945), friends (α = 0.958).

##### Perceived Stress Scale

The Perceived Stress Scale (PSS) is a 10-item self-report scale designed to measure the degree to which a person appraises situations as stressful (Cohen et al., 1983). It asks the respondent to rate their feelings and thoughts from the last month on a 5-point Likert-type scale from 0 (never) to 4 (very often). Total scores range from 0 to 40. Higher scores are indicative of greater overall perceptions of stress. The scale demonstrated excellent internal consistency in the present study (α = 0.902).

##### Depression Anxiety and Stress Scale

The Depression Anxiety and Stress Scale (DASS-21) is a self-report measure comprising three subscales: depression, anxiety and stress (Lovibond and Lovibond, 1995). There are seven items per domain, each rated on a 4-point Likert-type scale in terms of the severity/frequency at which they were experienced over the past week. Scores range from 0 (did not apply to me at all) to 3 (applied to me very much), and total scores range from 0 to 21, with higher scores indicating greater symptom severity. In the present study, internal consistency was good for each of the subscales: depression (α = 0.888), anxiety (α = 0.815), stress (α = 0.807).

##### Altman Self-Rating Mania Scale

The Altman Self-Rating Mania Scale (ASRM) is a short self-report measure assessing the presence and severity of manic symptoms in accordance with the *DSM*-IV criteria (Altman et al., 1997). The scale comprises five statement groups, and respondents must select one statement from each group that most applies to how they have felt over the past week. Each statement is rated from 0 (‘normal’ mood) to 4 (manic/hypomanic mood) and summed to form a total score from 0 to 20, with higher scores indicating greater mania severity (Altman et al., 1997). The scale has also demonstrated adequate reliability and good discriminant and concurrent validity (Altman et al., 1997). Internal consistency was good in the present study (α = 0.814).

##### Quality of Life in Bipolar Disorder Scale

Quality of Life in Bipolar Disorder Scale (QoL-BD) is a 56-item scale used to determine subjective quality of life in BP (Michalak and Murray; Collaborative RESearch Team to Study Psychosocial Issues in Bipolar Disorder [CREST.BD], 2010b). Items are rated on a 5-point Likert-type scale from 1 (strongly disagree) to 5 (strongly agree) according to each person’s experience over the past 7 days. Total scores range from 56 to 280 with higher scores indicating greater quality of life. The items span 12 domains: physical; sleep; mood; cognition; leisure; social; spirituality; finances; household; self-esteem; independence and identity, with higher scores indicating higher perceived quality of life. Test–retest reliability showed correlations of 0.69 (Michalak and Murray; CREST.BD, 2010b). In the present study, the scale demonstrated excellent internal consistency (α = 0.93).

#### Data analysis

**Figure 1.**
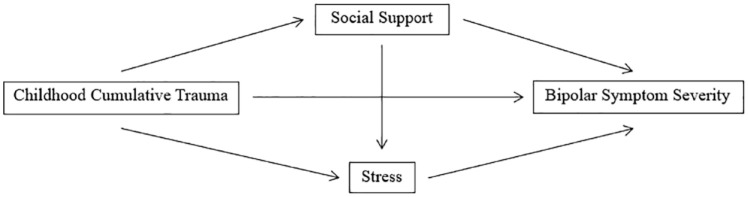
Predicted path diagram.

Multiple imputation was conducted using the MICE package on RStudio version 2021.09.2 for Windows. Data were then exported to SPSS Version 28 for analysis. Pearson’s *r* correlations were used to determine associations between predictor and outcome variables. Simultaneous multiple regression analysis was chosen to determine the predictive relationship of baseline reports of childhood cumulative trauma (CTQ), perceived social support (MSPSS) and perceived stress (PSS) to the following outcomes, assessed across self-report, interview or both: depression severity (MADRS and DASS depression subscale); suicidal ideation (MADRS item 10); mania severity (YMRS and ASRM); number of mood episodes (SCID-5-RV); anxiety severity (DASS anxiety subscale); overall quality of life (QoL-BD). Figure 1 presents the predicted path model for the relationship between childhood cumulative trauma, social support, stress and bipolar symptom severity (i.e. both depression and mania severity).

Childhood cumulative trauma was treated as a discrete variable with a range of 0 to 5, to reflect the number of types of traumas experienced during childhood, a method used in prior research (Cloitre et al., 2009), as measured by reaching the moderate to severe cutoff scores on any of the CTQ domains (emotional abuse, emotional neglect, sexual abuse, physical neglect and physical abuse) (Bernstein et al., 1998).

An a priori power calculation using G*Power indicated that a minimum sample of 77 would be required to detect a medium effect of the three predictors, using simultaneous multiple regression, with a power of 0.80 and α set to 0.05.

## Results

Participants included 114 people (*N* = 97; 85.1% female) living with BD-I (41.2%) or BD-II (58.8%). More than half of the participants were not in a relationship (59%) and were employed (57%) at the time of the study, with approximately two-fifths (39%) receiving government benefits. Approximately half of the participants (52%) had previously attempted suicide, and a little more than a third (35%) were hospitalised during their last mood episode. All demographic and clinical characteristics of the sample are presented in Table 1.

Exposure to childhood cumulative trauma was very high (*N* = 89; 78%), with the equal most common types of trauma exposure being emotional abuse (*N* = 83; 73%) and emotional neglect (*N* = 83; 73%), followed by sexual abuse (*N* = 72; 63%), physical neglect (*N* = 54; 47%) and physical abuse (*N* = 47; 41%). Pearson’s *r* correlations revealed that childhood cumulative trauma demonstrated a small negative correlation with social support (*r* [112] = −0.365, *p* < 0.01). Perceived social support demonstrated small negative correlations with perceived stress (*r* [112] = −0.337, *p* < 0.01), interviewer- and self-rated depression (*r* [112] = −0.27, *p* < 0.05; *r* [112] = −0.33, *p* < 0.01) and anxiety (*r* [112] = −0.242, *p* < 0.05), as well as a small positive correlation with quality of life (*r* [112] = 0.321, *p* < 0.01). Perceived stress demonstrated small positive correlations with suicidality (*r* [112] = 0.189, *p* < 0.01), total number of mood episodes (*r* [112] = 0.284, *p* < 0.05), interviewer-rated depression (*r* [112] = −0.365, *p* < 0.01) and a small negative correlation with quality of life (*r* [112] = −0.46, *p* < 0.01). Perceived stress also demonstrated moderate positive correlations with self-reported depression (*r* [112] = 0.501, *p* < 0.01) and anxiety (*r* [112] = 0.538, *p* < 0.01). Correlations for all predictor and outcome variables are presented in Table 2.

**Table 2.** Pearson’s *r* correlations for predictor and outcome variables, as well as variable inter-correlations.

	Childhood cumulative	Social support	Stress
MSPSS	–0.365^**^		
PSS	0.079	–0.337^**^	
MADRS	0.178	–0.27^*^	0.404^**^
DASS Depression	0.063	–0.333^**^	0.501^**^
YMRS	–0.063	0.062	0.107
ASRM	0.149	0.027	0.06
DASS Anxiety	0.065	–0.242^*^	0.538^**^
QoL-BD	–0.045	0.321^**^	–0.46^**^
MADRS item 10	–0.014	–0.001	0.189^**^
SCID-5-RV	–0.025	–0.029	0.284^*^

MSPSS: Multidimensional Scale of Perceived Social Support; PSS: Perceived Stress Scale; MADRS: Montgomery Asberg Depression Rating Scale; DASS: Depression Anxiety and Stress Scale; YMRS: Young Mania Rating Scale; ASRM: Altman Self-Rating Mania Scale; QoL-BD: Quality of Life in Bipolar Disorder Scale; SCID-5-RV: Structured Clinical Interview for *DSM*-5; *DSM*-5: *Diagnostic and Statistical Manual of Mental Disorders, Fifth Edition*.

*N* = 114.

* Statistical significance at *p* < 0.05.

** Statistical significance at *p* < 0.01.

Separate simultaneous multiple regression analyses were used to assess the ability of the three variables (childhood cumulative trauma [CTQ], perceived social support [MSPSS] and perceived stress score [PSS]) to predict the following illness outcomes: depression severity (MADRS and DASS depression subscale), suicidal ideation (MADRS item 10, score 1–6), mania severity (YMRS and ASRM), number of mood episodes over 6 months (SCID-5-RV), anxiety severity (DASS anxiety subscale) and overall quality of life (QoL-BD).

### Psychiatric symptoms

**Figure 2.**
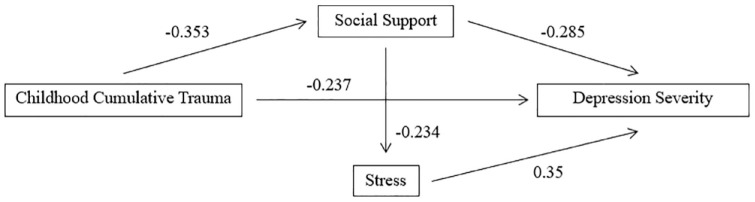
Actual path diagram for self-reported depression severity.

For interviewer-rated depression (MADRS) at baseline, a significant regression equation was found, *F* (3,110) = 9.795, *p* < 0.001, with the model accounting for 21.1% of the variance in depression severity. Only perceived stress (PSS) significantly contributed to the model (*p* < 0.001). At 6-month follow-up, the model remained significant, *F* (3,110) = 4.924, *p* = 0.003, accounting for 11.8% of the variance in interviewer-rated depression severity. Only perceived stress (PSS) significantly contributed to the model (*p* < 0.001). For self-reported depression (DASS) at baseline, a significant regression equation was found, *F* (3,110) = 222.55, *p* < 0.001, with the model accounting for 49.6% of the variance in self-reported depression severity. All three variables (childhood cumulative trauma [CTQ], perceived social support [MSPSS] and perceived stress score [PSS]) significantly contributed to the model. At 6-month follow-up, the model remained significant, *F* (3,110) = 13.339, *p* < 0.001, accounting for 26.7% of the variance in self-reported depression severity, with all three predictors making significant contributions to the model (the path diagram is presented in Figure 2).

For interview-rated suicidal ideation (MADRS item 10) at baseline, a significant regression equation was found, *F* (3,110) = 45.300, *p* < 0.001, with the model accounting for 16.7% of the variance in suicidal ideation at interview. All three predictors (childhood cumulative trauma [CTQ], perceived social support [MSPSS] and perceived stress score [PSS]) made significant contributions to the model. At 6-month follow-up, the regression equation was no longer significant.

**Figure 3.**
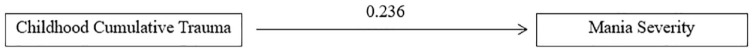
Actual path diagram for self-reported mania severity.

For interviewer-rated mania severity (YMRS), at baseline and 6-month follow-up, the regression equation was not significant. For self-reported mania (ASRM) at baseline, a significant regression equation was found, *F* (3,110) = 7.205, *p* < 0.001, with the model accounting for 3.1% of the variance in self-reported mania severity. Only childhood cumulative trauma (CTQ) and stress (PSS) were significant predictors. At 6-month follow-up, the model remained significant, *F* (3,110) = 2.815, *p* = 0.043, accounting for 7.1% of the variance in self-reported mania severity. Only childhood cumulative trauma (CTQ) made a significant contribution to the model (the path diagram is presented in Figure 3).

For total number of mood episodes (SCID-5-RV), a significant regression equation was found, *F* (3,110) = 12.243, *p* < 0.001, with the model accounting for 5.4% of the variance in the number of mood episodes experienced over the 6 months of the study. Only stress (PSS) made a significant contribution to the model. For self-reported anxiety (DASS anxiety subscale) at baseline, a significant regression equation was found, *F* (3,110) = 1829.682, *p* < 0.001, with the model accounting for 34.6% of the variance in self-reported anxiety severity. Only childhood cumulative trauma (CTQ) and stress (PSS) were significant predictors in the model. At 6-month follow-up, the model remained significant, *F* (3,110) = 11.916, *p* < 0.001, accounting for 24.5% of the variance in self-reported anxiety severity. Only stress (PSS) made a significant contribution to the model.

### Quality of life

In relation to quality of life (QoL-BD) at baseline, a significant regression equation was found, *F* (3,110) = 368.527, *p* < 0.001, with the model accounting for 62% of the variance in self-reported quality of life. All three predictors (childhood cumulative trauma [CTQ], perceived social support [MSPSS] and perceived stress score [PSS]) made significant contributions to the model. At 6-month follow-up, the model remained significant, *F* (3,110) = 11.331, *p* < 0.001, accounting for 23.6% of the variance in self-reported quality of life. Only social support (MSPSS) and stress (PSS) made significant contributions to the model.

All regression coefficients are presented in Tables 3 and 4 for interviewer-rated and self-reported outcomes, respectively.

**Table 3.** Regression coefficients for all models predicting clinical interview outcomes at baseline and 6-month follow-up.

Outcome	Predictors	Baseline					*p*	95% CI		6-Month follow-up					*p*	95% CI	
		R^2^	*t*	B	SE	β		Lower	Upper	R^2^	*t*	B	SE	β		Lower	Upper
Depression (MADRS)		0.21					<0.001^**^			0.12					0.003^*^		
	Childhood cumulative		–0.98	–0.60	0.62	–0.09	0.33	–1.82	0.62		0.93	0.58	0.62	0.09	0.35	–0.65	1.80
	Perceived social support		–1.81	–0.12	0.06	–0.17	0.072	–0.24	0.01		–0.79	–0.05	0.07	–0.08	0.43	–0.19	0.08
	Perceived stress		4.82	1.22	0.25	0.41	<0.001^**^	0.72	1.72		2.97	0.38	0.13	0.28	0.01^*^	0.13	0.63
Mania (YMRS)		0.02					0.53			0.01					0.9		
	Childhood cumulative		0.38	0.14	0.37	0.04	0.71	–0.59	0.87		0.21	0.06	0.30	0.02	0.83	–0.53	0.66
	Perceived social support		0.27	0.01	0.04	0.03	0.79	–0.07	0.09		–0.19	–0.01	0.03	–0.02	0.84	–0.07	0.06
	Perceived stress		1.42	0.11	0.08	0.14	0.16	–0.04	0.26		0.55	0.03	0.06	0.06	0.59	–0.09	0.15
Suicidal ideation (MADRS)		0.17					<0.001^**^			0.03					0.30		
	Childhood cumulative		–5.82	–0.21	0.04	–0.22	<0.001^**^	–0.28	–0.14		0.02	0.01	0.06	0.01	0.99	–0.11	0.12
	Perceived social support		–3.21	–0.01	0.01	–0.13	0.001^*^	–0.02	–0.01		1.01	0.01	0.01	0.12	0.32	–0.01	0.02
	Perceived stress		8.72	0.07	0.01	0.33	<0.001^**^	0.05	0.08		1.86	0.02	0.01	0.19	0.07	–0.01	0.05
Number of episodes (SCID)										0.06					0.09		
	Childhood cumulative										0.21	0.01	0.06	0.02	0.84	–0.11	0.14
	Perceived social support										0.16	0.01	0.01	0.02	0.88	–0.01	0.02
	Perceived stress										2.46	0.03	0.01	0.24	0.02^*^	0.01	0.06

CI: confidence interval; SE: standard error; MADRS: Montgomery Asberg Depression Rating Scale; YMRS: Young Mania Rating Scale; SCID: Structured Clinical Interview for *DSM*-5.

*N* = 114.

* Statistical significance at *p* < 0.05.

** Statistical significance at *p* < 0.001.

**Table 4.** Regression coefficients for all models predicting self-reported outcomes at baseline and 6-month follow-up.

Outcome	Predictors	Baseline					*p*	95% CI		6-Month follow-up					*p*	95% CI	
		R^2^	*t*	B	SE	β		Lower	Upper	R^2^	*t*	B	SE	β		Lower	Upper
Depression (DASS)		0.50					<0.001^**^			0.267					<0.001^**^		
	Childhood cumulative		1.98	1.23	0.09	0.06	0.048^*^	0.00	0.37		–2.70	–0.68	0.25	–0.24	0.008^*^	–1.19	–0.18
	Perceived social support		–5.36	–0.05	0.01	–0.17	<0.001^**^	–0.07	–0.03		–3.06	–0.09	0.03	–0.29	0.003^*^	–0.14	–0.03
	Perceived stress		21.39	0.41	0.02	0.62	<0.001^**^	0.37	0.44		4.03	0.21	0.05	0.35	<0.001^**^	0.11	0.31
Mania (ASRM)		0.03					<0.001^**^			0.07					0.043^*^		
	Childhood cumulative		2.88	0.30	0.10	0.12	0.004^*^	0.09	0.50		2.39	0.47	0.17	0.24	0.019^*^	0.08	0.87
	Perceived social support		1.56	0.02	0.01	0.07	0.12	–0.01	0.04		1.90	0.04	0.02	0.20	0.06	–0.01	0.08
	Perceived stress		–3.05	–0.06	0.02	–0.12	0.002^*^	–0.11	–0.02		1.68	0.07	0.04	0.16	0.097	–0.01	0.15
Anxiety (DASS)		0.35					<0.001^**^			0.245					<0.001^**^		
	Childhood cumulative		7.40	0.71	0.10	0.25	<0.001^**^	0.52	0.90		–0.44	–0.10	0.23	–0.04	0.663	–0.57	0.36
	Perceived social support		1.21	0.01	0.01	0.04	0.227	–0.01	0.03		–1.38	–0.04	0.03	–0.13	0.171	–0.09	0.02
	Perceived stress		16.25	0.32	0.02	0.54	<0.001^**^	0.28	0.36		5.01	0.24	0.05	0.44	<0.001^**^	0.14	0.33
Quality of life (QoL-BD)		0.62					<0.001^**^			0.24					<0.001^**^		
	Childhood cumulative		2.24	1.06	0.47	0.06	0.025^*^	0.13	1.99		0.79	1.44	1.82	0.07	0.43	–2.17	5.05
	Perceived social support		7.58	0.39	0.05	0.20	<0.001^**^	0.29	0.49		1.99	0.40	0.20	0.19	0.049^*^	0.00	0.79
	Perceived stress		–27.92	–2.70	0.10	–0.70	<0.001^**^	–2.89	–2.51		–4.49	–1.67	0.37	–0.40	<0.001^**^	–2.41	–0.94

CI: confidence interval; SE: standard error; DASS: Depression Anxiety and Stress Scale; ASRM: Altman Self-Rating Mania Scale; QoL-BD: Quality of Life in Bipolar Disorder Scale.

*N* = 114.

* Statistical significance at *p* < 0.05.

** Statistical significance at *p* < 0.001.

## Discussion

This study aimed to determine whether childhood cumulative trauma, perceived social support and perceived stress can be used to predict bipolar illness outcomes and quality of life. Consistent with the hypothesis, the combination of all three variables was able to predict baseline levels of depression severity (interviewer-rated and self-reported); mania severity (self-reported); suicidal ideation (interviewer-rated), anxiety severity (self-reported) and quality of life (self-reported). At 6-month follow-up, the model predicted depression severity (interviewer-rated and self-reported); mania severity (self-reported); anxiety severity (self-reported) and quality of life (self-reported).

In relation to depression, at 6-month follow-up, all three variables made significant unique contributions to self-reported depression severity; however, only stress contributed to interviewer-rated depression. The difference between self-reported and interviewer-rated depression is not unexpected, as these measures are related but not equivalent (Cuijpers et al., 2010), and self-report measures have previously demonstrated superiority in the prediction of treatment outcomes (Uher et al., 2012). The finding that social support is a unique predictor of depression severity in BD builds upon the prospective investigation of Cohen et al. (2004), who found that social support influenced depression recurrence over a 1-year follow-up among those with BD-I. Similarly, Johnson et al. (2000) highlighted the importance of social support in predicting depression at baseline and at 6-month follow-up in a BD-I sample. This finding is also in line with studies of childhood cumulative trauma on general population and BD samples that have demonstrated associations with depression symptoms (Agorastos et al., 2014; Martin et al., 2013; Sala et al., 2014; Suliman et al., 2009). The ability of perceived stress and childhood cumulative trauma to predict depression severity at 6-month follow-up is a novel finding of this study.

In relation to mania severity, childhood cumulative trauma was the only variable to make a significant unique contribution at 6-month follow-up but accounted for a small amount of the overall variance. This finding is in line with that of Sala et al. (2014) who found that the number of types of childhood trauma was related to more lifetime hypo/manic episodes over a 3-year follow-up. This finding is also in line with previous BD-I studies that have demonstrated manic recurrence cannot be predicted by stress (Cohen et al., 2004) or social support (Johnson et al., 2000). More research is needed to explore other predictors of mania recurrence that were not assessed in this study.

In relation to anxiety symptoms, only perceived stress made a significant unique contribution at 6-month follow-up. In research on the general population, stress and anxiety are known to have a bi-directional relationship, that is, increases in either one can exacerbate the other (Ray et al., 2017; Saccaro et al., 2021). These findings also support a recent investigation of people presenting to a general practitioner during the COVID-19 pandemic, which found that perceived stress was a significant predictor of anxiety severity (Ionescu et al., 2021).

In relation to quality of life, social support and stress were significant predictors at 6-month follow-up and explained the largest amount of variance of all outcome variables in this study. This finding aligns with the extensive support for associations between social support and quality of life in general population samples (Helgeson, 2003) and provides new evidence that social support plays an important role in QoL over time for those living with BD. This result is also consistent with a recent cross-sectional study that found higher levels of stress were associated with lower quality of life in a BD sample (Faurholt-Jepsen et al., 2019) and provides new evidence that stress predicts QoL over a 6-month follow-up. This finding is particularly important as both social support and perceived stress are modifiable factors that clinicians can target to improve wellbeing.

Taken together, these findings provide important insights for clinicians and researchers working with people with BD. Specifically, they highlight the need to collect a detailed trauma history and identify cases of exposure to multiple trauma types during childhood, as these individuals may be at risk of experiencing greater depression severity. In addition, those with BD experiencing heightened perceptions of stress or lower perceptions of social support over the previous 6 months may be more likely to experience greater severity of depression and anxiety and experience lower overall quality of life. Given that both social support and perceived stress were able to account for more of the variance across all outcomes at baseline than at 6-month follow-up, it suggests that current estimates of each will offer more reliable insights.

### Strengths

The strengths of this study include the use of a validated diagnostic interview to confirm BD diagnosis and to assess depression and hypo/mania severity as well as the number of mood episodes at each follow-up. In addition, the use of both self-report and interviewer-rated measures for depression and mania severity is another strength of this study. Finally, this is the first study to examine the combined and unique predictive ability of childhood cumulative trauma, perceived social support and perceived stress in determining bipolar illness outcomes and quality of life.

### Limitations and suggestions for future research

The conclusions that can be drawn from this study are limited by the small sample size and the relatively short follow-up period. There was also a high percentage of women in the sample, and findings may not be representative of all people living with BD. In addition, this study did not examine subsyndromal mood episodes, which may be better indicators of differences in the illness course among those who are not in the acute phase of the BD illness. Thus, more frequent assessment points and inclusion of subsyndromal symptom monitoring is needed. This study did not have sufficient resources to monitor and assess the impact of psycho- and pharmaco-therapy over the course of the study. It is likely that treatment received would influence the outcomes assessed, and this is a significant limitation of the present study. Future studies should consider assessing this. Future research should also evaluate programs aimed at increasing social support and managing stress, to determine their efficacy in improving illness outcomes and quality of life, particularly among those with BD and a history of childhood trauma.

## Conclusions

This study has demonstrated that the combination of childhood cumulative trauma, perceived social support and perceived stress is a strong predictor of quality of life, anxiety and depression severity in BD at baseline and 6-month follow-up. These findings are promising as social support and stress are modifiable factors which clinicians can target to improve outcomes for people living with BD. Future research should evaluate programs aimed at increasing social support and managing stress, to determine if this may assist in modifying the illness course and improve quality of life for those living with BD.
